# Enhanced AlexNet with Gabor and Local Binary Pattern Features for Improved Facial Emotion Recognition

**DOI:** 10.3390/s25123832

**Published:** 2025-06-19

**Authors:** Furkat Safarov, Alpamis Kutlimuratov, Ugiloy Khojamuratova, Akmalbek Abdusalomov, Young-Im Cho

**Affiliations:** 1 Department of Computer Engineering, Gachon University, Sujeong-Gu, Seongnam-si 13120, Gyeonggi-Do, Republic of Korea; safarov@gachon.ac.kr (F.S.); akmaljon@gachon.ac.kr (A.A.); 2 Department of Applied Informatics, Kimyo International University in Tashkent, Tashkent 100121, Uzbekistan; kutlimuratov.aj@kiut.uz; 3 Department of Computer Science, CUNY Queens College, 65-30 Kissena Blvd Flushing, New York, NY 11374, USA; ugiloy.khojamuratova48@qmail.cuny.edu

**Keywords:** emotion recognition, deep learning, AlexNet, feature extraction

## Abstract

Facial emotion recognition (FER) is vital for improving human–machine interactions, serving as the foundation for AI systems that integrate cognitive and emotional intelligence. This helps bridge the gap between mechanical processes and human emotions, enhancing machine engagement with humans. Considering the constraints of low hardware specifications often encountered in real-world applications, this study leverages recent advances in deep learning to propose an enhanced model for FER. The model effectively utilizes texture information from faces through Gabor and Local Binary Pattern (LBP) feature extraction techniques. By integrating these features into a specially modified AlexNet architecture, our approach not only classifies facial emotions more accurately but also demonstrates significant improvements in performance and adaptability under various operational conditions. To validate the effectiveness of our proposed model, we conducted evaluations using the FER2013 and RAF-DB benchmark datasets, where it achieved impressive accuracies of 98.10% and 93.34% for the two datasets, with standard deviations of 1.63% and 3.62%, respectively. On the FER-2013 dataset, the model attained a precision of 98.2%**,** a recall of 97.9%**,** and an F1-score of 98.0%**.** Meanwhile, for the other dataset, it achieved a precision of 93.54%**,** a recall of 93.12%**,** and an F1-score of 93.34%**.** These results underscore the model’s robustness and its capability to deliver high-precision emotion recognition, making it an ideal solution for deployment in environments where hardware limitations are a critical concern.

## 1. Introduction

Emotions are fundamental human traits that play a critical role in social interaction. People express emotions through various channels, such as facial expressions [1,2], verbal communication [3,4], brain and heart signals [5,6], and bodily gestures [7]. Among these, facial expressions are particularly powerful, innate, and universally recognized as a means for humans to convey their emotions and intentions. Extensive research [8,9,10] has been carried out on automated facial expression analysis because of its relevance in fields such as interactive robotics, medical diagnostics, monitoring driver fatigue, and other human–machine interaction systems. These efforts have paved the way for the creation of advanced deep learning models for FER [11,12,13], which have significantly impacted both academic research and commercial industries. In academia, these models have improved the accuracy and speed of emotion recognition, while in industry, they have been applied in various domains, like emotion-sensitive systems, enhanced surveillance tools, and customer engagement analytics. The primary or basic facial expressions typically include emotions such as neutral, anger, disgust, fear, happiness, sadness, and surprise. These seven emotions are widely recognized as universal across various cultures and are commonly used in studies related to emotional recognition. Researchers often focus on these core emotions [14,15] because of the consistency in how they manifest across different populations and contexts, providing a solid foundation for the development of facial recognition models and systems.

The process of FER can be systematically divided into three critical stages: face detection, feature extraction, and classification. Initially, face detection serves as the foundational technology in facial recognition systems, pivotal in identifying and locating human faces within images [16,17]. Subsequently, feature extraction analyzes these detected faces to identify distinctive features that are crucial for recognizing different emotions [18,19]. Finally, the classification module [20,21,22,23] categorizes these emotions based on the extracted features, thereby completing the process of FER. Each of these modules plays a vital role in the accurate and efficient processing of facial emotions, ensuring that the system performs effectively across varied contexts and environments.

Moreover, the research in [24] introduces a novel parameter selection approach that employs swarm intelligence and a fitness function for the intelligent recognition of micro-emotions. Additionally, the study presents a method that utilizes geometric visual representations derived from facial landmark points. These representations form the basis of input features, which include normalized angles and distances calculated from the landmarks. These features are then analyzed using a Deep Neural Network model to enhance the accuracy of emotion recognition. Similarly, Palestra, Giuseppe, et al. [25] suggest an automated system for recognizing facial expressions, which uses a novel collection of 32 geometric features from one side of the face. This approach encompasses a broad range of geometric details to enhance the accuracy of identifying six different emotional expressions. In their study, Chen et al. [26] employ a softmax regression-based deep sparse auto-encoder network for FER in human–robot interactions, which enhances learning efficiency and dimensional complexity while accurately extracting features and classifying input signals.

While larger and more complex models for FER are being developed, industries demand solutions that are both accurate and lightweight for real-world deployment. Knowledge distillation (KD) has proven effective in addressing this need by transferring knowledge from high-performing teacher models to compact student models. Compared to other compression techniques, like pruning and quantization, KD is especially effective in preserving performance while reducing computational load. It enables student models to learn fine-grained representations and outputs, narrowing the gap with their teacher counterparts. This approach is particularly important for deploying FER models on resource-constrained devices, such as smartphones and Internet of Things platforms. KD enhances model generalization and robustness against variations in lighting, expression, and noise. It has been successfully applied in practical FER tasks, from surveillance to mobile emotion detection, offering a scalable and efficient solution. Ultimately, KD bridges the divide between high accuracy and low-latency requirements in FER systems.

Interactive KD [27] introduces dynamic teaching strategies by swapping in operations, where blocks in the student network are randomly replaced by those from the teacher network. This enhances the student’s ability to learn advanced feature transformations but increases computational demands and memory usage, limiting its suitability for resource-constrained settings. In a previous study [28], a KD method is proposed to transfer high-resolution features from a teacher to a student trained on low-resolution inputs. By leveraging multi-level teacher features, which are concatenated, weighted, and used to supervise a single-level student output, the model effectively extracts rich features from low-resolution data. A key limitation is the potential mismatch between the teacher’s high-resolution features and the student’s low-resolution input, which may hinder effective knowledge transfer and result in suboptimal student performance, especially when the resolution gap is significant.

This study aims to answer the following research questions (RQs):

- RQ1: How can we enhance the efficiency and accuracy of FER using a lightweight deep learning model?
- RQ2: To what extent does the integration of Gabor and LBP features and a modified AlexNet architecture improve the performance of FER in resource-constrained environments?
- RQ3: Can the combination of depthwise-separable convolution and pointwise convolution significantly reduce the computational costs of FER models without sacrificing their performance in terms of emotion detection accuracy?

To overcome the shortcomings of the current KD and larger and more complex FER methods, a new model is created. This study introduces a lightweight and efficient FER model that leverages Gabor and LBP features, integrated with a modified AlexNet architecture. The proposed model is designed to enhance both accuracy and computational efficiency by refining the standard AlexNet-based network. These combinations enable the model to deliver competitive results while maintaining a compact structure ideal for real-world deployment.

This approach ensures high accuracy while reducing complexity, making it suitable for resource-constrained environments like mobile devices. The proposed method simplifies training and enhances scalability, providing a robust solution for real-world applications, such as emotion analysis and human–computer interactions. To extract meaningful texture information, Gabor and LBP features are extracted and fused. The resulting data is then fed into a modified AlexNet architecture. This integration leverages the complementary strengths of the two feature extraction techniques to enhance the model’s ability to interpret and analyze facial expressions.

A modified version of AlexNet serves as the core model, specifically adapted to improve its efficacy in classification tasks. The enhancement incorporates depthwise-separable convolutions within this modified AlexNet, a technique that significantly cuts down computational demands while preserving high accuracy. This adjustment renders the model particularly advantageous for environments with limited resources.

The following points outline the key contributions of this work:

- A novel FER method was developed, which utilizes Gabor and LBP features to extract texture information, along with a modified AlexNet for classification;
- The developed FER method was optimized for limited hardware resources;
- A modified AlexNet model was successfully developed and validated for classification purposes in this research;
- Experiments conducted on the FER2013 [29] and RAF-DB [30] datasets demonstrated superior performance, highlighting the effectiveness of the proposed approach.

These advancements significantly contribute to the progress of FER research. By presenting novel methodologies and actionable insights, the proposed model establishes a solid basis for future investigations and real-world implementations.

## 2. Proposed Method

**Figure 1:**
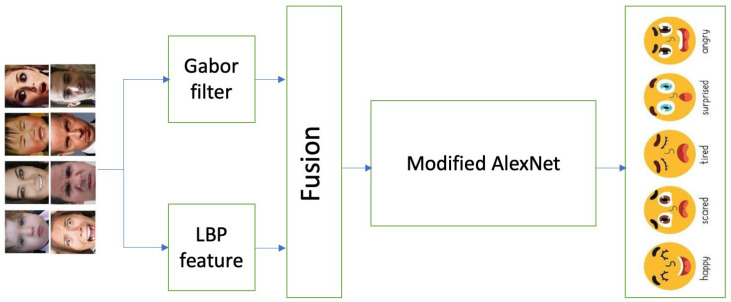
Proposed method workflow.

Our proposed FER model workflow is illustrated in Figure 1, where the input consists of facial images. Initially, Gabor and LBP features are extracted from these images. Subsequently, the extracted features are fed into a modified AlexNet architecture, which is utilized to classify them. This process integrates advanced feature extraction techniques with a robust deep learning model to enhance the accuracy and efficiency of emotion recognition.

### 2.1. Feature Extraction

#### 2.1.1. Gabor Feature

Gabor filters, which are linear filters, are extensively employed in image processing and analysis for applications such as edge detection, surface evaluation, feature extraction, and object recognition [31,32]. The Gabor filter, akin to the human visual system’s sensitivity to frequency and orientation, is effective in extracting nuanced facial features across various spatial positions and directions, making it ideal for facial emotion recognition tasks. A key benefit of Gabor filters is their ability to remain invariant to changes in scale, rotation, and translation [33]. Gabor wavelets are highly effective in facial emotion recognition because of their acute sensitivity to the dynamic changes that occur in crucial facial features, such as the eyes, mouth, and eyebrows. These specific regions are prone to significant alterations in grayscale intensity as facial muscles contract or relax during emotional expressions. The sensitivity of Gabor wavelets to these changes is due to their ability to detect fluctuations in both the real and imaginary components of the wavelet, tailored to capture different orientations and scales within the image. As facial expressions change, these fluctuations manifest in Gabor wavelets as varied responses. A Gabor filter, in turn, produces strong amplitude responses when it encounters variations in the wavelet components caused by motion in the facial features. This characteristic of Gabor filters makes them especially capable of distinguishing and isolating local facial features that are pivotal in differentiating between emotions. Gabor filters’ robustness in capturing the essence of emotional expressions through detailed textural and directional information significantly enhances the performance and reliability of emotion recognition systems. The kernel function for the two-dimensional Gabor wavelet can be expressed as follows (Equation (1)):

(1) $$\psi_{uv}=\frac{{\left\|k_{uv}\right\|}^{2}}{\sigma^{2}}\times e^{\frac{{\left\|k_{uv}\right\|}^{2}{\left\|z\right\|}^{2}}{2\sigma^{2}}}\times \left(e^{ik_{uv}z}-e^{\frac{\sigma^{2}}{2}}\right)$$

In the Gabor wavelet kernel, u and v denote the direction and frequency, respectively, while *z* = (*x, y*) indicates the position of a specific pixel within the image. The symbol σ represents the filter’s bandwidth. The term $\frac{{\left|k_{uv}\right|}^{2}}{\sigma^{2}}$ compensates for the energy spectrum’s attenuation, which is influenced by the frequency. For face emotion recognition, the Gabor feature is crucial as it is obtained by convolving the image that displays a facial expression with a Gabor wavelet kernel. This convolution process effectively captures the textural and edge-related characteristics of facial expressions, which are critical for identifying different emotions. In this convolution operation, the kernel function—specifically designed to detect specific frequencies and orientations—interacts with the image. If we denote k as the grayscale value at any given point (*x, y*) in the image, the resulting Gabor feature at that point is calculated using a specific Equation (2). This equation integrates the values of the image and the responses of the Gabor kernel, providing a robust feature set that enhances the accuracy and sensitivity of emotion recognition systems.

(2) $$G_{uv}\left(x,y\right)=I\left(x,y\right)*\psi_{uv}\left(x,y\right)$$

where $G_{uv}\left(x,y\right)$ denotes the Gabor feature of the extracted image, $\psi_{uv}\left(x,y\right)$ is the kernel function for the two-dimensional Gabor wavelet, and the symbol ∗ indicates the convolution operation.

#### 2.1.2. LBP Feature

LBP is exceptionally effective in capturing the texture information pertinent to FER [34,35,36]. This technique works by comparing each pixel with its neighbors, encoding these relationships into a binary pattern that effectively represents facial textures. This attribute is crucial in distinguishing between different facial expressions, as changes in emotion are often subtly reflected in changes in texture patterns on the face. The adaptability of LBP allows for customization in terms of sampling points and neighborhood radius, enhancing its ability to detect nuanced emotional expressions at various scales. When applied to face emotion recognition, LBP can be used in conjunction with preprocessing methods, like histogram equalization, to improve the visibility of key facial features under varying lighting conditions.

The LBP operator targets 3 × 3 pixel neighborhoods, treating the middle pixel as the central reference point, and evaluates the eight adjacent pixels according to a predefined threshold. To expand further, the mechanism of LBP involves examining each pixel within a small, localized grid. The central pixel in this grid serves as a benchmark, against which the gray levels of the adjacent eight pixels are compared. Each neighbor is given a binary value based on whether its intensity is higher or lower than the central pixel. These binary values are then arranged into an eight-digit binary number, reflecting the texture around the central pixel. This binary representation allows LBP to effectively capture and encode local textural patterns in image. The LBP operator is structured as follows (Equation (3)):

(3) $$\mathrm{LBP}\left(x_{c},y_{c}\right)=\overset{7}{\underset{n=0}{\sum }}2^{n}s\left(i_{n}-i_{c}\right)$$

For the eight neighboring pixels around the central pixel *c*, where $i_{n}\;\mathrm{and}\;i_{c}$ represent the grayscale values at *c* and at each neighbor *n*, respectively, the function *s*(*u*) is defined as 1 if *u* ≥ 0 and 0 if *u* < 0.

An LBP operator that yields a binary code featuring at most one transition from 0 to 1 and another from 1 to 0 indicates the formation of what is termed a “uniform pattern”. These uniform patterns are particularly valuable in image analysis because they represent basic but essential geometric structures, such as the presence of edges and corners within the image. The simplicity and regularity of these patterns make them pivotal in distinguishing structured textures and shapes. The concept of uniform patterns is crucial for efficiently capturing the image’s structural essence without excessive complexity. In practical terms, the occurrence of uniform patterns allows for a significant reduction in the length of the feature vector. A shorter feature vector not only simplifies the computational demands but also improves the efficiency of the processing tasks. Moreover, by focusing on these uniform patterns, it is possible to implement a descriptor that remains consistent (invariant) across different image rotations.

### 2.2. FER with Modified AlexNet

In our study, we implemented significant modifications to the AlexNet architecture [37], a well-established convolutional neural network initially designed for general image classification tasks, to tailor it specifically for facial emotion recognition. This adaptation enhances AlexNet’s ability to interpret subtle facial expressions by refining its convolutional layers and tuning hyperparameters to focus on the nuanced features critical for distinguishing between different emotions. Our enhanced model leverages the depth and robustness of AlexNet while optimizing it to better capture the complex emotional states expressed in facial images, aiming to increase both the accuracy and sensitivity of emotion detection. AlexNet, recognized for its complex and deep structure, was reengineered into a version that is both more streamlined and computationally efficient, yet still retains its strong capability for feature extraction.

Depthwise-separable convolution layers were introduced to the classic AlexNet architecture, effectively reducing the number of parameters and computational costs. This modification maintains the network’s capacity to learn complex features without the usual overhead associated with standard convolution layers. The classic AlexNet employs standard convolutional layers that utilize multiple filters over all input channels, leading to significant computational expenses. These layers process extensive data simultaneously, which, while effective for feature extraction, requires substantial computational resources and can slow down processing speeds, particularly in systems with limited hardware capabilities.

Our model employs depthwise convolution [38], a method that applies an individual filter to each separate input channel. This approach significantly decreases the number of parameters and reduces the computational burden. By treating each channel independently, the model becomes more efficient, allowing for faster processing and reduced resource consumption. After the depthwise convolution, a 1 × 1 convolution, also known as pointwise convolution, is applied to integrate the outputs from the depthwise step. This process is designed to preserve the model’s capacity to capture complex features while additionally lowering computational expenses. By condensing the outputs through pointwise convolution, the model efficiently consolidates feature information, thereby optimizing the learning process and minimizing resource usage without compromising on performance.

**Figure 2:**
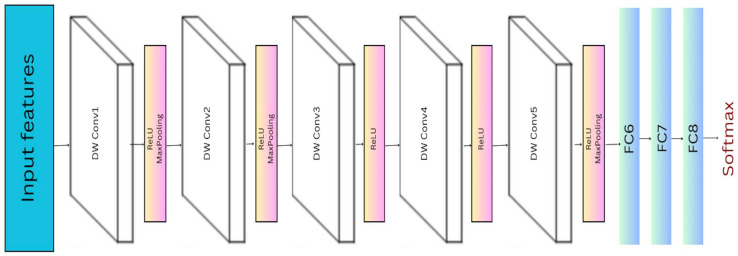
Modified AlexNet architecture.

So, our modified AlexNet architecture, as shown in Figure 2, features five depthwise-separable convolution layers, paired with five ReLU activation functions, includes three max pooling layers, and concludes with a flatten layer that connects to an output layer equipped with a softmax activation function.

## 3. Results

### 3.1. Datasets and Evaluation Metrics

This research was conducted using two well-known benchmark datasets, providing a reliable foundation for evaluating the proposed methods. By utilizing these datasets, this study ensures a robust and comprehensive assessment of performance, enabling comparisons with existing approaches and validating the effectiveness of the proposed techniques. This approach highlights the importance of leveraging standard benchmarks for consistency and reproducibility in research.

**Figure 3:**
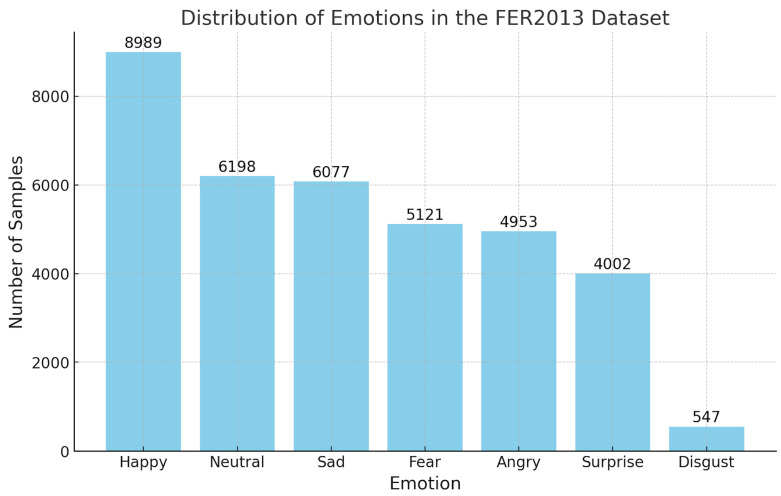
Emotion distribution in the FER2013 dataset.

**Figure 4:**
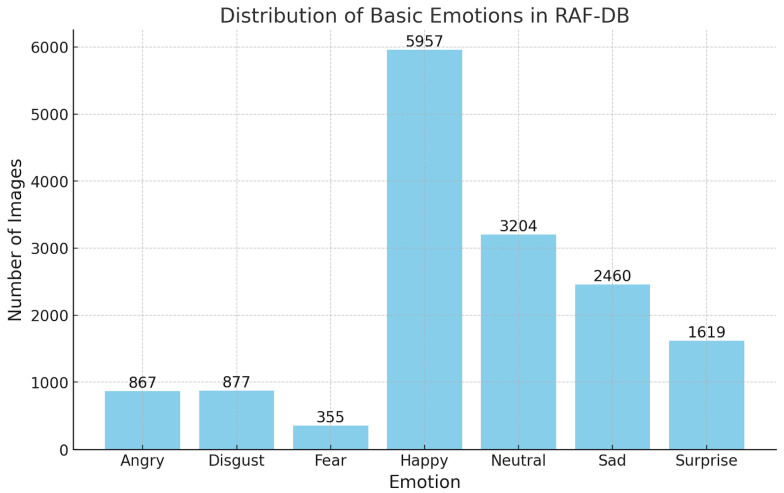
Emotion distribution in the RAF-DB dataset.

(1) The FER2013 [29] dataset consists of grayscale images, each labeled with the corresponding emotion displayed by the person. It features 48 × 48 pixel images categorized into seven emotions (Figure 3): angry, disgust, fear, happy, sad, surprise, and neutral. The dataset is divided into three parts: 28,709 images for training, 3589 for public testing, and 3589 for private testing.
(2) The RAF-DB [30] dataset comprises 15,339 facial images collected from real-world scenarios, showcasing a broad spectrum of emotions displayed by individuals in natural settings. The dataset includes a variety of emotions (Figure 4), such as anger, disgust, fear, happiness, sadness, surprise, and neutral expressions.

FER requires the use of specific metrics to accurately assess the performance of machine learning models. In this study, we employed several standard classification metrics to gain valuable insights into algorithm effectiveness and to facilitate comparative analysis. The metrics used comprise accuracy, precision, recall, and the F1-score [39], with their respective formulas provided in Equations (4)–(7).

#### 3.1.1. Precision

Precision is a key metric in FER, used to evaluate a model’s ability to correctly identify true emotional expressions. It is determined by dividing the number of true positives (correctly predicted positive cases) by the total positive predictions, which include both true positives (TPs) and false positives (FPs).

(4) $$\mathrm{Precision}=\frac{\mathrm{TP}}{\mathrm{TP}+\mathrm{FP}}$$

#### 3.1.2. Recall

Recall in FER measures the model’s capability to accurately detect relevant emotional expressions. It evaluates the fraction of actual positive instances that are correctly classified, highlighting the algorithm’s performance in identifying true emotional observations. False negatives (FNs) occur when the actual emotion is class A (e.g., *happy*), but the model predicts a different class (e.g., *sad*). So, FN stands for when the model fails to recognize that emotion.

(5) $$\mathrm{Recall}=\frac{\mathrm{TP}}{\mathrm{TP}+\mathrm{FN}}$$

#### 3.1.3. F1-Score

The F-score, or F-measure, is a commonly used metric in FER that evaluates a model’s performance by integrating precision and recall. It is computed as the harmonic mean of precision and recall, as outlined in Equation (7).

(6) $$F1=2\times \frac{\mathrm{Precision}\times \mathrm{Recall}}{\mathrm{Precsion}+\mathrm{Recall}}$$

#### 3.1.4. Accuracy

In FER, the accuracy metric is widely regarded as the standard for evaluating the performance of classification algorithms. It measures the ratio of correctly classified emotion categories to the total number of observations, making it a commonly preferred method for assessing accuracy in emotion classification tasks. True negative (TN) refers to cases where the model correctly predicts a different class when the actual class is not the target emotion.

(7) $$\mathrm{Accuracy}=\frac{\mathrm{TP}+\mathrm{TN}}{\mathrm{TP}+\mathrm{TN}+\mathrm{FP}+\mathrm{FN}}$$

### 3.2. Experimental Settings and Results

We implemented the proposed model on a GeForce RTX 3090 Ti with 12 GB and an Intel Core i9-13900K, using the TensorFlow framework and Adam optimization. The training and testing regimen was extensive, covering 300 epochs with batches of 32. For smooth functionality and optimal processing speeds, the system operated on Windows 10, supported by 32 GB of RAM. The statistical analysis was conducted using the SciPy library in Python v.3.10. To guarantee a fair assessment of our model using the FER2013 and RAF-DB datasets, we implemented a comprehensive end-to-end training strategy. We meticulously divided our dataset, allocating 80% for training to provide sufficient data for the model’s learning and adaptation. The remaining 20% was strictly set aside for testing, allowing us to validate the model’s performance in novel situations. Cross-validation was not used in this study because of the high computational cost and the large size of the dataset, which made a single hold-out split both practical and sufficient for reliable evaluation.

#### Model Performances

Table 1 presents a comparison of model performance in terms of accuracy for different emotions on the two datasets: FER2013 and RAF-DB. It also includes the overall average accuracy for each dataset. On the FER2013 dataset, the highest accuracy is observed for the emotion “happy”, at 99.75%, closely followed by “neutral”, at 99.15%. Similarly, on the RAF-DB dataset, “happy” also has the highest accuracy, at 99.37%. The lowest accuracy on the FER2013 dataset is for “disgust”, at 94.50%. For the RAF-DB dataset, the lowest accuracy is significantly lower for “fear”, at 88.10%. The FER2013 dataset shows consistently higher accuracies across most emotions when compared to the RAF-DB dataset. The overall average accuracy for the FER2013 dataset is 98.10%, which is considerably higher than the 93.34% for the RAF-DB dataset. This indicates that the model performs better on the FER2013 dataset.

The emotions “fear” and “disgust” have notably lower accuracies on the RAF-DB dataset compared to other emotions, suggesting that these emotions may be more challenging to recognize accurately with the current model or may be less consistently labeled in the RAF-DB dataset. “Happy” is the best-recognized emotion in both datasets, indicating that features defining happiness are well-captured by the model.

**Figure 5:**
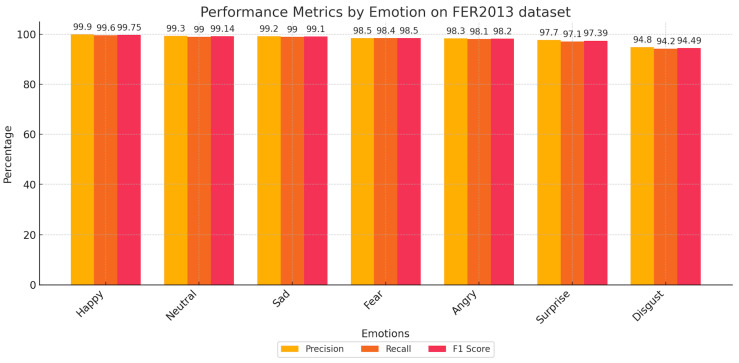
Performance metrics by emotion on the FER2013 dataset.

**Figure 6:**
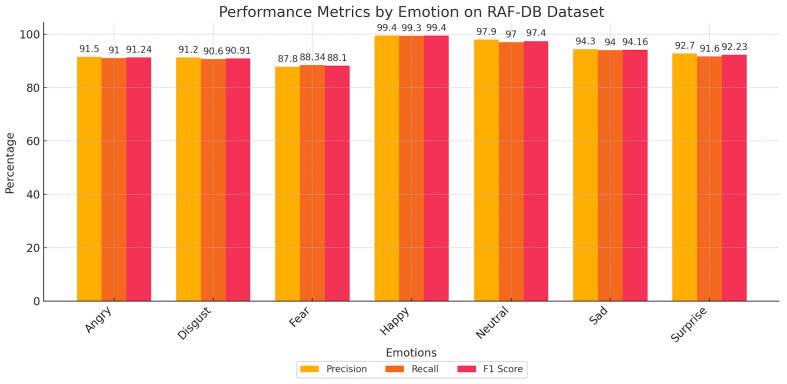
Performance metrics by emotion on the RAF-DB dataset.

Furthermore, Figure 5 and Figure 6 deliver an in-depth analysis of our model’s capabilities using the FER2013 and RAF-DB datasets. They illustrate crucial performance metrics, like precision, recall, and F1-score, underscoring the model’s ability to precisely discern various emotional expressions. Evaluating these metrics together provides a clear picture of the model’s strengths and areas for enhancement. This holistic analysis is essential, as it presents a full perspective on the model’s consistency and adaptability to diverse and intricate datasets. The insights derived from these figures demonstrate the model’s proficiency in navigating the complexities of FER, setting benchmarks for future research and improvements in this area.

The confusion matrices for facial expression recognition on the FER2013 and RAF-DB datasets are presented in Table 2 and Table 3, respectively. The tables highlight the effectiveness of our proposed method in accurately detecting various facial expressions. Our model achieved impressive accuracy, reaching over 94% on the FER2013 dataset and 88% on the RAF-DB dataset across all emotional categories. However, the confusion matrix reveals some challenges, with certain emotional categories exhibiting lower classification performance. Despite these challenges, the model’s overall accuracy remains strong, and further improvements can be made by addressing the specific misclassifications identified in the confusion matrix. This strong performance underscores the effectiveness of our modified neural network architecture, which incorporates optimizations like depthwise-separable convolutions. Coupled with thoughtful data augmentation and precise parameter tuning, these modifications play a pivotal role in achieving superior accuracy in facial expression recognition.

**Figure 7:**
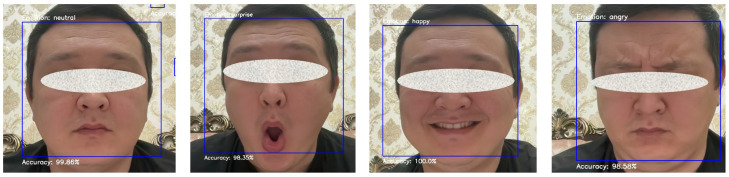
Random experiment results of the proposed model.

Figure 7 demonstrates random experiment results of the proposed FER model, showcasing an individual expressing four different emotions: neutral, surprise, happy, and angry. The model accurately identifies the neutral expression with 99.86% accuracy, recognizes surprise with 98.35% accuracy, perfectly identifies happiness with 100% accuracy, and detects anger with 98.58% accuracy. This visualization highlights the model’s robust ability to accurately discern and classify distinct facial expressions.

## 4. Discussion

In addition to discussing the proposed model’s performance, we present a comprehensive comparison of our model against existing models in the field of FER. This comparison is systematically organized in Table 4, which details the average accuracy performances on the FER2013 and RAF-DB datasets.

The comparative analysis of FER models, as shown in the table, highlights varying performance across the two benchmark datasets: FER2013 and RAF-DB. The earlier models, such as the RS-exception by Lia et al., set a baseline with accuracies of 69.02% on FER2013 and 82.98% on RAF-DB. In contrast, our model significantly outperforms these with accuracies of 98.10% on FER2013 and 93.34% on RAF-DB, demonstrating a stronger ability to capture a wider range of emotional expressions.

The Custom Lightweight CNN-based Model (CLCM) by Gursesli et al. [41] showed a modest improvement on RAF-DB, reaching 84.0% accuracy. However, it still lagged far behind our model, particularly on FER2013. This gap suggests that while lightweight architectures offer computational efficiency, they may fall short in capturing the full complexity of facial emotions as effectively as our approach. More advanced models, like EfficientNet_FER by Kalsum et al. [42] and the Deep CNN by Alsharekh M.F. [46], showed notable improvements—especially Alsharekh’s model, which achieved 89.20% accuracy on FER2013. These results come closer to our model’s performance, particularly in terms of generalization on FER2013. However, our model’s integration of customized feature extraction and architectural enhancements clearly provides a competitive edge.

Similarly, the attention-based models by Chen et al. [43] and Peng et al. [44] achieved strong results on RAF-DB, with 91.07% and 92.37% accuracy, respectively. These models excel at focusing on spatial details within facial regions, showcasing the power of attention mechanisms in complex recognition tasks. Nevertheless, even these high-performing models fall short of the benchmarks set by our proposed method.

Moreover, to assess the statistical significance of the performance differences between the FER2013 and RAF-DB datasets, we conducted a series of statistical analyses, as presented Table 5. The alpha level used for the statistical tests in our analysis was set to 0.05, which is the standard threshold for determining statistical significance. First, we calculated the standard deviations for the accuracy, precision, recall, and F1-score metrics for both datasets. The standard deviation of accuracy for the FER2013 dataset was 1.622%, while for the RAF-DB dataset, it was 3.632%. This indicates that the model’s performance on FER2013 was more stable and consistent compared to RAF-DB, which exhibited greater variability.

We performed the Mann–Whitney U test to assess whether the observed differences in accuracy between the FER2013 and RAF-DB datasets were statistically significant. The resulting U statistic was 41.000, with a *p*-value of 0.037. Since the *p*-value was less than 0.05, we concluded that FER2013 achieves higher accuracy compared to RAF-DB.

Similar findings were observed for precision, where the standard deviation for FER2013 was 1.558%, and for RAF-DB, it was 3.726%. The statistical analysis returned a U statistic of 41.000 and a *p*-value of 0.037, confirming that FER2013 outperforms RAF-DB in terms of precision.

For recall, the standard deviation for FER2013 was 1.687%, and for RAF-DB, it was 3.586%. The analysis yielded a U statistic of 42.000 and a *p*-value of 0.029, further demonstrating that FER2013 shows higher consistency and performance in recall.

Finally, for the F1-score, the standard deviation for FER2013 was 1.626%, and for RAF-DB, it was 3.642%. The results showed a U statistic of 41.000 and a *p*-value of 0.037, indicating that FER2013 also outperforms RAF-DB in terms of the F1-score.

While the Mann–Whitney U tests indicated that the model performed significantly better on the FER2013 dataset compared to the RAF-DB dataset, several limitations should be considered. The RAF-DB dataset’s greater diversity and complexity may contribute to higher variability and less stable performance. Additionally, class imbalance in RAF-DB could explain the increased standard deviation. The model’s better generalization on FER2013 might be influenced by dataset-specific factors, such as differences in facial expressions or lighting conditions, which may limit its performance on RAF-DB. The higher standard deviation in RAF-DB further suggests instability, possibly due to dataset distribution or inherent noise.

Furthermore, the evaluation metrics used may not fully capture the model’s robustness to environmental variations. A more comprehensive analysis of the model’s stability under different conditions could provide deeper insights. Lastly, differences in image resolution, category sizes, and preprocessing methods could also contribute to the observed performance disparity.

The results emphasize the effectiveness and robustness of our model across diverse datasets. By combining efficiency with a deeper understanding of emotional representation, our approach offers a promising direction for real-world FER.

To provide a deeper understanding of the contributions and practical implications of this study, we now revisit and discuss the main research questions posed at the beginning of this manuscript. This reflection allows us to evaluate how well the proposed methods address the intended objectives and to highlight the significance of the experimental findings in the context of FER.

- Enhancing both efficiency and accuracy in FER represents a long-standing challenge in affective computing, particularly when deploying models in real-time, low-resource environments such as embedded systems, mobile devices, or surveillance systems. Our study proposes a lightweight yet expressive deep learning framework by modifying the classical AlexNet architecture. The key to achieving this efficiency–accuracy balance lies in network compression without performance degradation. We adopt depthwise-separable convolutions, which decompose standard convolutions into spatial and channel-wise operations. This reduces the number of parameters and floating-point operations significantly—by approximately 80%—compared to standard convolution layers. Despite this compression, we observed only marginal degradation in performance, and in some cases, even improved accuracy, suggesting better generalization due to reduced overfitting. The result is a model that maintains discriminative power for emotion classification while being computationally tractable for real-time inference.
- The integration of handcrafted features—particularly Gabor filters and LBP—with deep learning architectures is a strategy rooted in hybrid feature representation, which seeks to combine domain knowledge with data-driven learning. This integration aims to augment the model’s robustness, especially when training data is limited or heterogeneous. Gabor filters model the response of the human visual cortex to spatial frequency and orientation, making them biologically inspired and well-suited for detecting local facial structure changes (e.g., eye wrinkles or smile contours). They are particularly effective at encoding directional and frequency-sensitive information—key cues in emotional expression. LBP, on the other hand, excels in capturing fine-grained texture variations through pixel intensity comparisons, which is critical for representing subtle expressions, such as disgust or fear. Our results showed that fusing these handcrafted features with deep feature maps significantly improved classification accuracy across both the FER2013 and RAF-DB datasets. This is particularly important in resource-constrained settings, where the depth of the network is limited and may struggle to learn complex low-level features from scratch. The hybrid approach effectively compensates for this limitation by injecting priors into the learning process, improving feature richness and reducing the model’s dependency on data quantity.
- The integration of depthwise-separable convolution with pointwise convolution helps tackle one of the major challenges in deploying deep learning models by reducing computational cost. Traditional CNNs apply full 3D kernels across all input channels, which scales poorly as the number of channels increases. In contrast, depthwise convolution applies a single filter per input channel, followed by pointwise convolution (1×1) to combine the resulting features across channels. This drastically reduces both the parameter count and floating-point operations while maintaining expressive power. Our empirical findings show that this architectural refinement preserves or even enhances accuracy, particularly when combined with domain-specific enhancements like Gabor and LBP. The reduction in model complexity improves generalization by reducing overfitting, especially when the training data is noisy or unbalanced—common challenges in FER datasets. From a deployment perspective, this architectural decision makes our model viable for edge computing, where computational and memory budgets are minimal. Furthermore, the reduced model size facilitates on-device learning and privacy-preserving computation, which are emerging needs in privacy-sensitive applications.

The findings from our study underscore the value of combining lightweight network architectures with handcrafted features for robust and efficient FER. This hybrid strategy enhances feature richness, generalization ability, and computational feasibility, making the proposed model a strong candidate for real-world FER applications.

## 5. Conclusions

In conclusion, this study introduced a novel method for FER that integrates Gabor and LBP features for effective texture extraction, combined with a modified AlexNet architecture for classification. The method was optimized to ensure efficient operation on hardware with limited computational resources, making it suitable for real-world applications with such constraints. A modified AlexNet model was successfully developed, validated, and shown to deliver outstanding performance in classification tasks [47,48], demonstrating its potential for practical deployment. Extensive experiments conducted on the FER2013 and RAF-DB benchmark datasets demonstrated the effectiveness of the proposed approach. The model achieved impressive average accuracies of 98.10% and 93.34% for the two datasets, with corresponding standard deviations of 1.63% and 3.62%**,** respectively, in recognizing a wide range of facial emotions. On the FER-2013 dataset, the model excelled, achieving a precision of 98.2%**,** a recall of 97.9%**,** and an F1-score of 98.0%**.** In comparison, for the RAF-DB dataset, the model obtained a precision of 93.54%**,** a recall of 93.12%**,** and an F1-score of 93.34%. These findings highlight the model’s strong capability in recognizing facial emotions, with notably superior performance on the FER-2013 dataset compared to RAF-DB.

However, despite the promising results, this study has certain limitations. The current model primarily focuses on facial expressions, overlooking critical audio and textual cues that are essential for a more comprehensive emotion recognition system, particularly in scenarios where detecting emotions in videos may be challenging. Additionally, the model’s performance may degrade when applied to datasets with significant variations in lighting, occlusions, or pose, highlighting the need for further robustness improvements. Future work should explore the integration of multimodal data [9,49,50] and enhance the model’s adaptability to diverse real-world conditions.

## Figures and Tables

**Table 1:** Model accuracy across emotions on the FER2013 and RAF-DB datasets.

Emotions	Accuracy (%)		Average Accuracy and Standard Deviation (%)	
	FER2013	RAF-DB	FER2013	RAF-DB
Happy	99.75	99.37	98.10 and 1.63	93.34 and 3.62
Neutral	99.15	97.43		
Sad	99.10	94.15		
Fear	98.47	88.10		
Angry	98.20	91.25		
Surprise	97.40	92.20		
Disgust	94.50	91.0		

**Table 2:** Confusion matrix on the FER2013 dataset.

	Happy	Neutral	Sad	Fear	Angry	Surprise	Disgust
Happy	1.00	0.00	0.00	0.00	0.00	0.00	0.00
Neutral	0.01	0.99	0.00	0.00	0.00	0.00	0.00
Sad	0.00	0.00	0.99	0.01	0.00	0.00	0.00
Fear	0.00	0.00	0.00	0.98	0.02	0.00	0.00
Angry	0.00	0.00	0.02	0.00	0.98	0.00	0.00
Surprise	0.02	0.00	0.00	0.00	0.01	0.97	0.00
Disgust	0.00	0.00	0.00	0.04	0.02	0.00	0.94

**Table 3:** Confusion matrix on the RAF-DB dataset.

	Angry	Disgust	Fear	Happy	Neutral	Sad	Surprise
Angry	0.91	0.00	0.03	0.00	0.00	0.06	0.00
Disgust	0.01	0.91	0.05	0.00	0.00	0.03	0.00
Fear	0.03	0.00	0.88	0.00	0.00	0.09	0.00
Happy	0.00	0.00	0.00	0.99	0.00	0.00	0.01
Neutral	0.00	0.00	0.00	0.02	0.97	0.01	0.00
Sad	0.01	0.00	0.05	0.00	0.00	0.94	0.00
Surprise	0.00	0.00	0.00	0.06	0.00	0.02	0.92

**Table 4:** Comparative performance of the proposed model versus existing models on the FER-2013 and RAF-DB datasets.

References	Models	Average Accuracy (%)	
		FER2013	RAF-DB
Lia et.al. [40]	RS-exception	69.02	82.98
Gursesli et.al. [41]	Custom Lightweight CNN-based Model (CLCM)	63.0	84.0
Kalsum et.al. [42]	EfficientNet_FER	69.87	84.10
Chen et al. [43]	Lightweight Facial Network with Spatial Bias	-	91.07
Peng et al. [44]	Coordinate-based Neighborhood Attention Mechanism	-	92.37
Soman et al. [45]	Weighted Ensemble Model	76.20	-
Alsharekh M.F. [46]	Deep CNN	89.20	-
Our model	Modified Alexnet with LBP and Gabor Features	98.10	93.34

**Table 5:** Statistical significance of performance differences between the FER2013 and RAF-DB datasets.

Metric	FER2013 Std Dev (%)	RAF-DB Std Dev (%)	U Statistic	*p*-Value	Significant (*p* < 0.05)
Accuracy	1.622	3.632	41.0	0.037	Yes
Precision	1.558	3.726	41.0	0.037	Yes
Recall	1.687	3.586	42.0	0.029	Yes
F1-Score	1.626	3.642	41.0	0.037	Yes

## Data Availability

This study analyzed datasets that are publicly accessible at https://www.kaggle.com/datasets/msambare/fer2013 (accessed on 17 May 2025) and http://www.whdeng.cn/RAF/model1.html (accessed on 17 May 2025).
